# Mean Field Games for Diel Vertical Migration with Diffusion

**DOI:** 10.1007/s11538-023-01154-3

**Published:** 2023-04-27

**Authors:** Maksim Mazuryn, Uffe Høgsbro Thygesen

**Affiliations:** Department of Applied Mathematics and Computer Science, Technical University of Denmark, Asmussens Allé, 303B, 2800 Kgs. Lyngby, Denmark

**Keywords:** Vertical migration, Optimal behaviour, Mean field games, Habitat selection, 91A16, 92D50, 49N80, 49N20

## Abstract

We present a theoretical framework, based on differential mean field games, for expressing diel vertical migration in the ocean as a game with a continuum of players. In such a game, each agent partially controls its own state by adjusting its vertical velocity but the vertical position in a water column is also subject to random fluctuations. A representative player has to make decisions based on aggregated information about the states of the other players. For this vertical differential game, we derive a mean field system of partial differential equations for finding a Nash equilibrium for the whole population. It turns out that finding Nash equilibria in the game is equivalent to solving a PDE-constrained optimization problem. We detail this equivalence when the expected fitness of the representative player can be approximated with a constant and solve both formulations numerically. We illustrate the results on simple numerical examples and construct several test cases to compare the two analytical approaches.

## Introduction

Mean field game theory, introduced by Lasry and Lions (2007), models games with a large number of interacting agents in the limit as the number of players goes to infinity $N \rightarrow +\infty$. To make the limiting case tractable, it is usually assumed that all the players are equivalent, meaning that they share the same set of admissible strategies and have identical structure of the utility function, but see for example (Carmona and Zhu 2016; Bensoussan et al. 2018) for games with relaxed assumption on the symmetry between the players. Every agent has access to aggregated information about the organization of the population and has to make decisions based on the mean field structure of the available information about the surrounding environment. In turn, individual decisions of each agent change the macroscopic organization of the whole population. Mean field game theory has a number of applications in economic theory and financial engineering (Carmona 2020; Gomes et al. 2015; Bertucci et al. 2020), behaviour of crowds (Carmona and Delarue 2018a) and policy design (Carmona 2016).

Another domain with large populations of interacting agents is ecology, where animals may interact through predation and compete for limited resources. One example of a game with a vast number of players is diel vertical migration in aquatic systems, which is believed to constitute the largest movement of biomass on the planet and is ubiquitous in the ocean (Brierley 2014). In the migration process animals remain in deep water layers during daylight hours to avoid visual predators and migrate to upper levels in a water column at dusk to feed.

This manuscript contributes to a series of papers to understand and model diel vertical migration using a game theoretical framework. Seminally, Iwasa (1982) constructed a matrix game model of the phenomenon, which was later elaborated by several authors. Sainmont et al (2013) divided the water column in two layers—namely surface and deep layers, but noted also that the identified Nash equilibrium is not evolutionarily stable due to the absence of self-interaction; see also Gabriel and Thomas (1988). Pinti and Visser (2019) increased the spatial resolution but kept the time resolution in two epochs, viz. day and night. These models use coarse resolution in either space or time (or both), thus giving a very crude approximation of the positions and strategies of the players at transition time period—at dawn and dusk. Thygesen and Patterson (2019) modelled vertical migrations in continuous space and time, using control theory tools (Liberzon 2011) for finding optimal strategies of the animals. This allows to resolve the narrow time frame at dawn and dusk and a more direct comparison to data. However, the resulting modelling framework is more technical and Thygesen and Patterson (2019) ignored the costs and constraints of locomotion. Thygesen and Mazuryn (2022) resolved the dynamics in continuous space and time taking into account the cost of motion, but the equations are limited to the shallow water case, where the optimal density of the players is strictly positive at each point of a water column. The aim of this manuscript is to extend our previous modelling framework from Thygesen and Mazuryn (2022) by adding random fluctuations in the vertical position of the agents to cover all depth ranges. In contrast to the previous paper, we utilize the Feynman–Kac formula (Kac 1949) to derive the resulting mean field system, so that our previous model is a special case of the new one.

Thus, we model diel vertical migration as a mean field game with a continuum of individuals and apply the mean field game theoretical framework to describe Nash equilibrium in terms of partial differential equations. We add random fluctuations to the state of each agent which might be due to incomplete perception of the surrounding environment or random forces acting on the individual, e.g. from turbulence. It is important to stress that any two agents have independent trajectories of the white noise realizations. The case with correlated noise, common noise or shocks (Carmona and Delarue 2018a, b) will not be considered in this manuscript.

An important contribution of this work is establishing two equivalent formulations of the problem: As a system of partial differential equations via mean field limit or, under quasi-static approximation, as a PDE-constrained optimization problem. This equivalence allows access to a wide range of analytical tools to study the problem analytically and numerically.

## Method

In this section, we start with formulation of the vertical differential game on the individual level, where modelling parameters are introduced and discussed. Due to symmetrical interaction between the players, the population-level formulation of the vertical game is derived via the mean field limit and utilizing the Feynman–Kac formula. Moreover, it turns out that the Nash equilibrium in the game can also be found through solving PDE-constrained optimization, which is derived and discussed in the last section.

### The Vertical Game Formulation

We consider a population of a generic species distributed in a water column [0, *H*] of maximum depth *H* with $X_t^{(i)}$ as the vertical position of *i*th player at time *t*. Our model includes only vertical migration of the agents and disregards horizontal dynamics. We model diel vertical migration of marine organisms as a game with infinitely many players where each animal plays against others, seeking regions with high concentration of food and low mortality subject to cost of motion. The vertical speed $V_t^{(i)}$ can be considered as a strategy the *i*th player can choose to play against other agents. Sometimes we will utilize control theory terminology and refer to $X_t^{(i)}$ and $V_t^{(i)}$ as a state of the individual and its control, respectively.

It is assumed that all the players are identical, and the information an agent has access to is a mean field type, i.e. each player can only see aggregated information about the population. Due to the symmetry between the players, we choose a representative player to model the behaviour of the whole population. To highlight indistinguishability of the agents in the game, we omit upper indexes in the state $X_t$ and the control $V_t$ variables and consider them as vertical coordinate and speed of the representative player, respectively.

The position dynamics of the representative agent in the water column is driven by the stochastic differential equation:

1 $$\begin{aligned} \mathop {}\!\textrm{d}{X}_t = V_t \mathop {}\!\textrm{d}{t} + \sigma \mathop {}\!\textrm{d}{B}_t, \end{aligned}$$

with $B_t$ as standard Brownian motion scaled with constant noise level $\sigma$, and each player in the population has an independent realization of the Brownian motion. The scaling factor $\sigma$ is the same for all the players.

Equation (1) describes vertical motion of the representative player who can control its vertical speed $V_t$ at each moment of time subject to uncontrolled external random fluctuations $\sigma \mathop {}\!\textrm{d}{B}_t$ of its vertical position. We impose reflective boundary condition on the surface and the bottom of the water column [0, *H*] assuming that no new agents can appear through the boundary.

Each player in this vertical game optimizes the fitness functional over its lifetime period:

2 $$\begin{aligned} J(V) = \mathbb {E}^{X_0 = x} \left[ \int _0^{\tau } g(X_s, s) - \frac{\nu }{2} V^2(X_s, s) \mathop {}\!\textrm{d}{s} \right] , \end{aligned}$$

where $\tau$ is a random time of death of the representative player. For the process $X_t$ to be alive means that for $t < \tau$, it follows Eq. (1), while for time $t \ge \tau$ after the “death”, we assume that the process enters a special “coffin” state $X_t = \partial$ and stays there for $t \ge \tau$ (Oksendal 2013). The killing rate is defined as:

$$\begin{aligned} \mu (x, t) = \lim _{s \rightarrow 0} \frac{1}{s} \mathbb {P}^{X_t = x} \left[ X_{t+s} = \partial \right] , \quad x \ne \partial \end{aligned}$$

Further in the text, we use terms killing rate and risk rate interchangeably.

The functional (2) represents the fitness of the representative player defined as the expected accumulated energy of the player over the lifetime period following the trajectory $X_t$ and the vertical speed $V_t$, which are coupled by the stochastic differential equation (1). This expression of the functional favours regions of high food concentration with the energy harvest rate *g* and avoids visiting regions with high mortality rate $\mu$, which is implicitly present through the death time $\tau$ of the agent. The functional takes into account the cost of locomotion by penalizing quick changes in the vertical position of the player. We assume that the animals are small enough, for example copepods or other zooplankton, to keep the cost of motion proportional to the squared vertical speed with drag coefficient $\nu$.

The risk rate $\mu$ is defined as:

3 $$\begin{aligned} \mu (x, t) = \mu _0(x, t) + \mu _1 N(x, t) . \end{aligned}$$

The risk rate is composed of density-independent mortality rate $\mu _0$, which represents the risk of encounter of a single agent with visual predators and defined as a function of light abundance, and the density-dependent mortality term $\mu _1 N$ which penalizes aggregation of the players. The latter term in (3) is a mean field component of the model which describes the interaction between the representative player and the whole population.

We fix the time period $T = 24$ h and assume that both harvest *g* and density-independent mortality $\mu _0$ rates are periodic functions with a period *T*, i.e. all days are identical and seasonal fluctuations are disregarded. The selected time period is small enough to neglect population dynamics and fluctuations in the population size. We fix the total biomass in the water column and normalize it to 1. Now, the optimal density *N* can be considered as a probability distribution of the player’s vertical position at a given point of time.

Utilizing the above-mentioned symmetry assumption, we are looking for the vertical speed as a Markovian closed-loop control in the form $V = V(X_t, t)$, i.e. the optimal strategy for the representative player is a function of its state $X_t$ at time *t*. With periodic mortality $\mu _0$ and harvest *g*, we are looking for a time-periodic solution pair (*N*, *V*) to the differential game.

### Mean Field System

The outlined formulation of the game as a control problem with the state (1) and the objective functional (2) can be transformed into a system of partial differential equations via the mean-field limit. The idea is to utilize the postulated symmetry of the information and the fact that the game has infinite number of players. It allows to zoom out from the individual-level dynamics of the representative player described by the stochastic differential equation (1) and consider the time evolution of the population density.

The stochastic differential equation (1), which governs evolution of the vertical position of the representative player, is consistent with the forward Kolmogorov or Fokker–Plank equation:

4 $$\begin{aligned} \frac{\partial N}{\partial t} = - \frac{\partial (N V)}{\partial x} + \frac{\sigma ^2}{2} \frac{\partial ^2 N}{\partial x^2}. \end{aligned}$$

We omit mortality and source terms in the forward Kolmogorov equation from the random death time $\tau$ because we are solving the vertical game in a short time period, relative to the lifetime of the players, where the population dynamics can be disregarded.

The no-flux condition on the boundary $x = \{0, H \}$ of the water column is expressed as:

5 $$\begin{aligned} N V - \frac{\sigma ^2}{2} \frac{\partial N}{\partial x} = 0. \end{aligned}$$

The normalization condition for the total biomass is written as:

$$\begin{aligned} \int _0^H N(y, t) \mathop {}\!\textrm{d}{y} = 1, \end{aligned}$$

for time $$t \in [0, T]$$.

We now define the value function *U* for a given density *N* as the expected fitness of the representative player starting in $X_t = x \ne \partial$ at time *t* and who plays optimally:

6 $$\begin{aligned} U(x, t) = \sup _V \mathbb {E}^{X_t = x} \left[ \int _t^{+ \infty } \left( g(X_s, s) - \frac{\nu }{2} V^2(X_s, s) \right) e^{-\int _t^s \mu (X_{\xi }, \xi ) \mathop {}\!\textrm{d}{\xi }} \mathop {}\!\textrm{d}{s} \right] . \end{aligned}$$

Here, the random trajectory $\{X_s\}$ depends both on the initial condition $X_t=x$ and on the strategy *V*. The first part of the integrand coincides with the fitness expression from (2). The exponential scaling in the integrand represents the probability that the player survives from *t* to *s*, which allows us to replace the upper limit $\tau$ in (2) with $\infty$ (Thygesen 2022). We aim to find the optimal velocity field *V* which maximizes the expected fitness of the representative player.

The integral in the expression (6) exists by the dominated convergence theorem (Rudin et al 1976) because both terms *g* and *V* are bounded functions and the integral in the exponent is a nonnegative bounded number. Invoking the Feynman–Kac formula (Kac 1949; Thygesen 2022), the fitness *U* from (6) is the solution to:

7 $$\begin{aligned} \frac{\partial U}{\partial t} + \sup _{v} \left( LU + g - \frac{\nu v^2}{2} \right) = 0, \end{aligned}$$

where the generator *L* is defined as the differential operator:

$$\begin{aligned} LU = v \frac{\partial U}{\partial x} + \frac{\sigma ^2}{2} \frac{\partial ^2 U}{\partial x^2} - \mu U. \end{aligned}$$

As the function inside the supremum in (7) is a concave quadratic function of the argument *v*, the global maximum exists and is attained at $V = \frac{1}{\nu } \frac{\partial U}{\partial x}$.

The value function *U* must satisfy a homogeneous Neumann boundary condition at the boundary $x\in \{0,H\}$:

$$\begin{aligned} \frac{\partial U}{\partial x}(x, t) = 0 \quad \text {for} \quad x \in \{0, H \} . \end{aligned}$$

Mathematically, this boundary condition follows from the duality between the forward and backward Kolmogorov equations, here (4) and (7): Together with the no-flux condition (5), it ensures that boundary terms vanish when computing the time derivative of the total fitness $\int N(x,t) U(x,t) ~\mathop {}\!\textrm{d}{x}$, so that there is no flux of fitness over the boundary (Thygesen 2022). More heuristically, the homogeneous Neumann condition states that there is no fitness gradient at the boundary, because the diffusive reflection at the boundary would immediately attenuate such a gradient. From the point of view of an individual animal which happens to be located at a boundary which is a fitness minimum, the vanishing fitness gradient implies that this animal should not exercise any effort to actively move away from the boundary, but rather wait for the diffusion to do the initial displacement.

The time interval of our interest for the control problem is [0, *T*] due to the periodicity assumption. The boundary condition for the value function on the interval is $U(x, 0) = U(x, T)$, meaning that the expected fitness of the representative player at $t = 0$ is the same as at $t = T$, assuming that the player survives during the one day time interval.

Substituting the form for the optimal velocity *V* into the Hamilton–Jacobi–Bellman equation, we arrive to the following mean field system:

8 $$\begin{aligned} {\left\{ \begin{array}{ll} \frac{\partial N}{\partial t} = - \frac{\partial (N V)}{\partial x} + \frac{\sigma ^2}{2} \frac{\partial ^2 N}{\partial x^2} &{} (x, t) \in (0, H) \times (0, T) \\ \frac{\partial U}{\partial t} + \frac{1}{2 \nu } \left( \frac{\partial U}{\partial x} \right) ^2 + \frac{\sigma ^2}{2} \frac{\partial ^2 U}{\partial x^2} + g - U (\mu _0 + \mu _1 N) = 0 &{} (x, t) \in (0, H) \times (0, T) \\ \int _0^H N(y, t) \mathop {}\!\textrm{d}{y} = 1 &{} t \in [0, T] \\ U(x, 0) = U(x, T) &{} x \in [0, H] \\ N(x, 0) = N(x, T) &{} x \in [0, H] \\ (N V - \frac{\sigma ^2}{2} \frac{\partial N}{\partial x})(x, t) = 0 &{} x \in \{0, H \} \\ \frac{\partial U}{\partial x}(x, t) = 0 &{} x \in \{0, H \} \\ V = \frac{1}{\nu } \frac{\partial U}{\partial x} &{} \end{array}\right. } \end{aligned}$$

The PDE system (8) is similar to the one from (Thygesen and Mazuryn 2022) with a couple of major differences: First, there is a diffusion term in the Fokker–Plank equation due to the randomness in the vertical position of the representative player. Next, system (8) includes a partial differential equation in the value function *U* (viz., the Hamilton–Jacobi–Bellman equation) rather than in the velocity field *V* itself. Finally, since we do not impose the quasi-static approximation for the value function, the mortality term in the Hamilton–Jacobi–Bellman equation includes *U* instead of a constant approximation of the expected fitness.

Our modelling set-up is not covered by the contemporary existence and uniqueness theorems in the mean field game theory literature (Lasry and Lions 2007; Carmona 2016; Carmona and Delarue 2018a, b). Therefore, we use the verification theorem approach for the Hamilton–Jacobi–Bellman equation (Oksendal 2013): If we can solve the PDE system (8), then we have found a Nash equilibrium of the vertical game, and the optimal control exists and indeed is given by $V = \frac{1}{\nu } \frac{\partial U}{\partial x}$. Numerical analysis will reveal if the solution is locally unique, i.e. if the differential equations are locally well-posed.

### PDE-Constrained Optimization Formulation

In this section, we aim to show that the Nash equilibrium in the differential mean field game can also be found by optimizing over the population, although the mortality term in this equivalent optimization problem has a slightly different form. This equivalence provides flexibility in the choice of numerical methods for finding vertical strategies and distributions: We can use optimization-based methods for solving a PDE-constrained optimization problem rather than equation-solving methods for the system (8). Similar observations on equivalence between differential games and PDE-constrained optimization have been made (Lasry and Lions 2007); here, we detail the argument because our form of the cost functional is slightly different, and also to keep the presentation self-contained.

Consider the PDE-constrained optimization problem:

9 $$\begin{aligned} \max _{N, V} \int _0^H \int _0^T \left( g - F \left( \mu _0 + \frac{\mu _1 N}{2}\right) - \frac{\nu V^2}{2}\right) N \mathop {}\!\textrm{d}{s}\mathop {}\!\textrm{d}{y}, \end{aligned}$$

where the parameter *F* describes fitness, as will be detailed in the following. The maximization is over all pairs (*N*, *V*) which satisfy the PDE constraint given by the forward Kolmogorov equation:

10 $$\begin{aligned} \frac{\partial N}{\partial t} = - \frac{\partial (N V)}{\partial x} + \frac{\sigma ^2}{2} \frac{\partial ^2 N}{\partial x^2} . \end{aligned}$$

In addition, we apply the boundary conditions from the PDE system (8) as constraints, viz. zero flux at the boundaries $x\in \{0,H\}$, and time periodicity $N(x,0)=N(x,T)$. We also assume normalization, $\int _0^H N(x,t) ~\mathop {}\!\textrm{d}{x} = 1$. The objective function can be viewed as a total energy gain of the population over a day, where the parameter *F* converts loss of individuals into a loss of energy.

We approach this PDE-constrained optimization problem with the method of Lagrange multipliers (De los Reyes 2015). Specifically, the Lagrange relaxed objective functional is

11 $$\begin{aligned} L(N, V, p) =&\left\langle g - F \left( \mu _0 + \frac{\mu _1 N}{2}\right) - \frac{\nu V^2}{2}, N \right\rangle \nonumber \\&- \left\langle \frac{\partial N}{\partial t} + \frac{\partial (N V)}{\partial x} - \frac{\sigma ^2}{2} \frac{\partial ^2 N}{\partial x^2} , p \right\rangle , \end{aligned}$$

where *p* is a Lagrange multiplier corresponding to the constraint (10). The brackets here indicate inner product, i.e. integrals over both space and time.

We now pursue stationarity conditions by calculating functional derivatives of the Lagrange functional *L* with respect to each of the variables *N*, *V* and *p* along admissible directions $\delta N$, $\delta V$ and $\delta p$. At the optimum, these derivatives must vanish. With repeated use of integration by parts, we obtain:

12 $$\begin{aligned} L_N(N, V, p) \delta N= & {} \left\langle g - F (\mu _0 + \mu _1 N) - \frac{\nu V^2}{2} \right. \nonumber \\{} & {} \left. + \frac{\partial p}{\partial t} + \frac{\partial p}{\partial x} V + \frac{\sigma ^2}{2} \frac{\partial ^2 p}{\partial x^2}, \delta N \right\rangle \nonumber \\{} & {} - \oint _{ \partial \Omega _{t = \{0, T\} }} p \delta N \cdot \varvec{n} \mathop {}\!\textrm{d}{\alpha } - \frac{\sigma ^2}{2} \oint _{\partial \Omega _{x = \{0, H\} }} \frac{\partial p}{\partial x} \delta N \cdot \varvec{n} \mathop {}\!\textrm{d}{\alpha }\nonumber \\= & {} 0. \end{aligned}$$

13 $$\begin{aligned} L_V(N, V, p) \delta V= & {} \left\langle N \left( \frac{\partial p}{\partial x} -\nu V \right) , \delta V \right\rangle = 0. \end{aligned}$$

14 $$\begin{aligned} L_p (N, V, p) \delta p= & {} -\left\langle \frac{\partial N}{\partial t} + \frac{\partial (N V)}{\partial x} - \frac{\sigma ^2}{2} \frac{\partial ^2 N}{\partial x^2}, \delta p \right\rangle = 0. \end{aligned}$$

These equalities should hold in any admissible direction $\delta N$, $\delta V$ and $\delta p$. Thus, the expression (14) recovers the forward Kolmogorov equation (10), which also appears in the mean field system (8). From (13), we arrive to $V = \frac{1}{\nu } \frac{\partial p}{\partial x}$, which establishes the relation between the adjoint state *p* and the velocity field *V*, similar to the relationship between the value function *U* and the optimal control *V* in (8). The inner product in (12) vanishes for any perturbation $\delta N$ if *p* satisfies the PDE

15 $$\begin{aligned} \frac{\partial p}{\partial t} + \frac{\partial p}{\partial x} V + \frac{\sigma ^2}{2} \frac{\partial ^2 p}{\partial x^2}+ g - F (\mu _0 + \mu _1 N) - \frac{\nu V^2}{2} = 0. \end{aligned}$$

Note in particular that the quadratic term $\langle F \mu _1 N/ 2, N\rangle$ in (11) gives rise to the term $F \mu _1 N$, which explains why the factor 1/2 was included in (9). The contour integrals vanish when the co-state *p* is a periodic function of time and satisfies the homogeneous Neumann boundary condition for $x\in \{0, H\}$.

The PDE (15) needs not have a time-periodic solution *p*. Taking inner product of the left side in (15) with *N*, and assuming that *p* is time-periodic, we find that

$$\begin{aligned} \left\langle N,g - F (\mu _0 + \mu _1 N) - \frac{\nu V^2}{2} \right\rangle = 0 \quad \text{ so } \quad F = \frac{ \left\langle N,g - \frac{\nu V^2}{2} \right\rangle }{ \left\langle N, \mu _0 + \mu _1 N \right\rangle }. \end{aligned}$$

This particular value of *F*, which ensures a periodic solution *p*, can be viewed as the expected energy gain of an organism over its remaining lifetime, assuming that this lifetime is large compared to the single day so that temporal fluctuations can be neglected. This is consistent with our interpretation of “fitness”. For other values of *F*, the optimization problem is still well-posed but will lead to a gain or loss of fitness over the day. This can happen when the fitness is not in equilibrium, for example due to parameters varying on the slower time scale of seasons. Such an imbalance is most easily addressed by including a Lagrange multiplier on the normalization constraint that *N* integrates to 1; this step is detailed in Thygesen (2022) for the much simpler time-invariant and density-independent case ($\mu _1=0$).

Thus, equation (15) differs from the Hamilton–Jacobi–Bellman equation governing *U* in (8) in two respects, which both concern the mortality: First, in (8), the mortality is multiplied with the value function *U*(*x*, *t*), while in (15), the mortality is multiplied on the constant fitness *F*. With the previous discussion in mind, we can view *F* as a spatiotemporal average of the value function *U*, and the difference between the two disappears in the low-mortality limit where animals expect to live much longer than a single day in a truly periodic environment, so that spatiotemporal fluctuations in *U* become negligible. Second, the density-dependent mortality in the mean field game (8) has coefficient $\mu _1$, while the coefficient in (15) is $\mu _1/2$. This is a reminder that in a mean field game, the agents tend to their own interests and do not pursue the common good. We will elaborate on this in the discussion.

It has been shown (Lasry and Lions 2007) that a wider class of differential games can be recast as (modified) PDE-constrained optimization problem, viz. those where the running cost can be written as a functional derivative of some potential (De los Reyes 2015). Our system is a special case in this sense, but has the additional feature of mortality and periodicity.

The constrained optimization problem (9) optimizes over all admissible pairs (*N*, *V*). These pairs can (for example) be parametrized in terms of the vertical velocity field *V*, because the nonzero diffusivity parameter $\sigma$ implies that there exists a unique density *N* which satisfies the forward Kolmogorov equation for a given *V*.

## Numerical Study

In this section, we take the system of PDEs (8) and solve it numerically for different parameter scenarios. Specific forms of the harvest and mortality rates are taken from our previous paper and here we briefly present essential parts of the model to make the text self-contained. For a more detailed discussion on the model parameters, we refer to Thygesen and Mazuryn (2022). The model is inspired by the vertical migration of zooplankton subject to visual predators like planktivorous fish. It will be shown that daily fluctuations in the light levels at depth cause diel vertical migration of the animals.

### Model Set-Up

The mortality rate $\mu _0$ corresponds to the chance of being detected and eaten by a predator, a planktivorous fish. The predator detects its prey by vision, so $\mu _0$ depends on light levels as detailed in the following. The detection criterion is that enough photons scattered from a prey arrive on the predator’s eye and trigger a neural response. From the point of view of the prey, the risk of encounter is the probability that a predator is closer than a critical radius *r*, where detection can take place. If predators cruise through the environment, the prey’s rate of being detected is proportional to the cross section area of the so-called detection sphere, i.e. a sphere with radius *r*.

The illumination at the surface is a periodic function with a period *T*:

$$\begin{aligned} I_s(t) = \frac{I_0}{1 + \exp \left( A \cos (2\pi t/T) \right) } \end{aligned}$$

At depth in the water column, the light intensity decays exponentially with the absorption coefficient $k_1$:

$$\begin{aligned} I(x, t) = I_s(t) \exp (-k_1 x) \end{aligned}$$

According to the visual range model with saturation (Aksnes and Utne 1997), the detection radius satisfies the equation

$$\begin{aligned} r^2 \exp (k_1 r) = \gamma ^2 \frac{I(x, t)}{K + I(x, t)} \end{aligned}$$

which takes into account the local light intensity *I*, the spherical spread of light from prey to predator, and light absorption along this path. The parameter $\gamma$ here combines the reflection properties of the prey with the visual sensitivity of the predator eye, and describes the detection distance in clear water ($k_1=0$) at high light intensity ($I=\infty$).

Then, the risk rate $\mu _0$ is proportional to the cross-section area of the detection sphere, i.e. to $r^2$. We let *m* denote the scaling constant and add a constant baseline mortality $\mu _{\text {base}}$, which is due to other causes than predation:

$$\begin{aligned} \mu _0(x, t) = m r^2 + \mu _{\text {base}}. \end{aligned}$$

The energy harvest rate *g* is proportional to the food abundance in the environment—phytoplankton concentration for this specific example. For the sake of simplicity, to avoid explicit modelling phytoplankton distribution in the sea, we assume that the harvest rate is time invariant and depends logistically on depth:

$$\begin{aligned} g(x, t) = \frac{g_0}{1 + \exp (k_2 (x - x_{\text {clin}}))}. \end{aligned}$$

**Table 1:** Model parameters mostly from Thygesen and Mazuryn (2022) with added diffusivity and baseline mortality

Symbol	Parameter	Value	Units
*H*	Maximum depth	500	m
*T*	Period	24	h
$I_0$	Maximum irradiance	1	$\textrm{W m}^{-2} \textrm{nm}^{-1}$
*K*	Saturating light level	1	$\textrm{W m}^{-2} \mathrm {nm^{-1}}$
$\gamma$	Maximum detection distance	0.02	$\textrm{m}$
$k_1$	Absorption coefficient	0.02	$\textrm{m}^{-1}$
*A*	Amplitude of light fluctuations	13	–
$\nu$	Drag	$10^{-6}$	$\mathrm {J ~h ~ m}^{-2}$
$\sigma ^2$	Diffusion	20	$\mathrm {m^2~ h}^{-1}$
$\mu _1$	Density-dependent mortality	1	$\textrm{h}^{-1}$
*m*	Mortality scaling	100	$\textrm{m}^{-2} ~ \textrm{h}^{-1}$
$\mu _{\text {base}}$	Baseline mortality	$10^{-5}$	$\textrm{h}^{-1}$
$k_2$	Harvest decay rate	0.02	$\textrm{m}^{-1}$
$g_0$	Maximum food availability	0.01	$\textrm{J} ~ \textrm{h}^{-1}$
$x_{\text {clin}}$	Mixed layer depth	100	$\textrm{m}$

### Numerical Methods

To calculate numerical solutions, we discretize the system (8) with a spectral element scheme using the FEniCS package Alnæs and Wells 2015. The computational domain $[0, H] \times [0, T]$ is triangulated and the analytical solution is approximated by polynomials with pre-defined degrees on each triangle. In calculations, we select first- and second-order polynomials to approximate the unknown function pair (*N*, *V*).

The discretized version of the PDE system is solved by a Newton-type method where we can control the step size. This approach is different from that in Thygesen and Mazuryn (2022), where we developed a homotopy method and solve a sequence of auxiliary problems to find a Nash equilibrium in the vertical game.

### Numerical Results

**Fig. 1:**
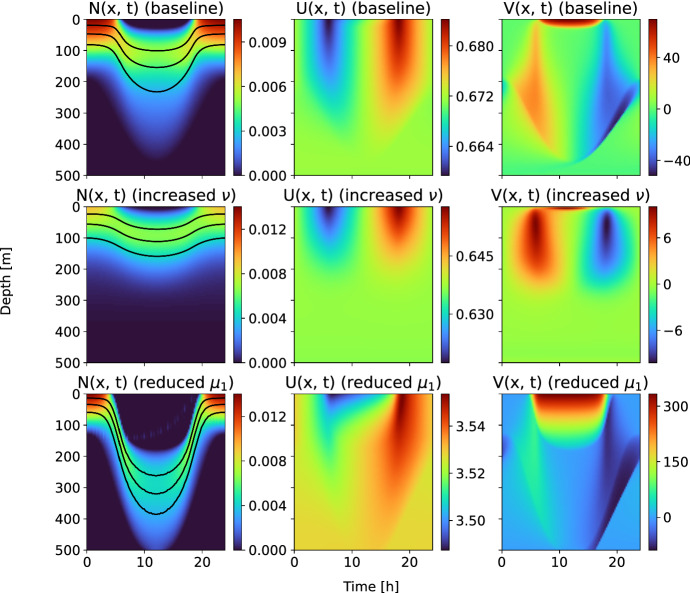
(Color figure online) Nash equilibrium for the model. *Left panels*: Optimal density *N*. *Middle panels*: Value function *U*. *Right panels*: Vertical velocity field *V*. *Top row*: Baseline case with the parameters from Table 1. *Middle row*: Increased cost of motion $\nu$ ($\times 10$). *Bottom row*: Reduced density-dependent mortality $\mu _1$ ($\times 0.1$)

We take the parameters from Table 1 and numerically solve the system of partial differential equations (8). Figure 1 (top panels) displays the optimal distribution *N* of the population in the water column for the fixed time period with the optimal vertical velocity field *V*. Black lines correspond to mean trajectories of three individual players, computed as quantiles in the vertical distributions.

During daytime hours the animals move to deep water layers with low light intensity to reduce the encounter rate with the visual predators as in Fig. 1 (top left panel). When light intensity decreases at dusk, the players return to upper layers with decreased mortality risk near the surface to benefit from higher nutrient concentration. It should be noted that the optimal density *N* is symmetric around $t = 12$ h which, as expected, comes from the symmetry of the model parameters.

Naively, one would anticipate a symmetric velocity profile *V* by looking at the symmetry of the optimal density in Fig. 1 (top right panel). In contrast to the density *N*, the vertical speed field is not an odd function. The model predicts that at around $t=10$, animals descend faster than they ascend at around $t=16$, and it is due to high risk of being close to the surface during daytime. The speed distribution shows that if an agent gets in that region by influence of the error $\sigma \mathop {}\!\textrm{d}{B}_t$ in the vertical position, then it should leave the region immediately even at a cost of high speed.

The origin of the asymmetry comes from the state dynamics (1) of the representative player. Two processes contribute to the final position of the agent: the player can only control the advection part by choosing appropriate vertical speed, while the diffusion process is left uncontrolled. The agent tries to compensate contribution of the diffusion part to the position in the water column by adjusting its vertical speed. We refer to “Appendix A” for a more analytical discussion on the observed asymmetry in Fig. 1 and numerical study of the analytical model for various noise levels $\sigma$.

Figure 1 also displays optimal densities *N*, value functions *U*, and velocities *V* for modified model parameters (second and third rows). In the second row, the cost of motion has been increased by multiplying the drag parameter $\nu$ with a factor 10. As expected, now the players are less inclined to move and stay in the preferred water level. In the bottom row, the risk rate $\mu _1$ describing density-dependent mortality has been decreased by a factor 10, which can correspond to decreasing predator abundance in the water column. Reduced number of predators makes beneficial high aggregation of the players. Now, the fitness is increased (right figure), and, therefore, the animals not only aggregate more, but also go deeper during the daytime to avoid the risky region near the surface with high probability of being seen by the predators.

### Numerical Comparison of the MFG System and the PDE-Constrained Problem

In this section, we examine the equivalence between the mean field system (8) and the PDE-constrained optimization problem (9) from a numerical viewpoint. To compare these methods, we construct two test cases where fluctuations in the value function *U* are small enough to examine corresponding numerical approximations. The quasi-static approximation of the value function *U* allows comparison between the mean field system and the PDE-constrained optimization problem.

**Fig. 2:**
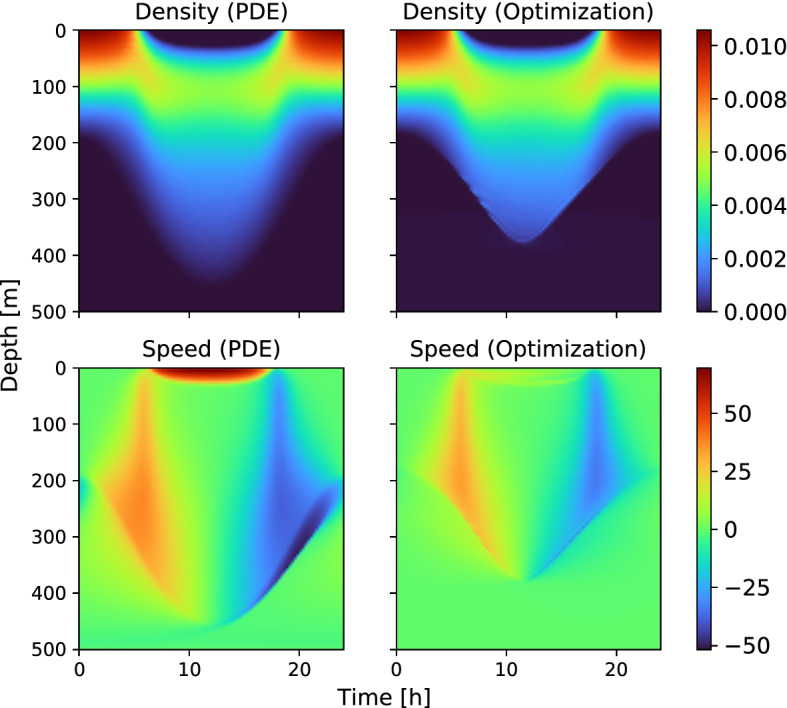
(Color figure online) Nash equilibrium for the parameters from Table 1. *Left column*: Mean field PDE system. *Right column*: PDE-constrained formulation

For the first example, we take the model parameters from Table 1 and find the corresponding numerical solutions for the optimal density *N* and the optimal velocity *V*. Numerical approximations of the solution are shown in Fig. 2.

Note that the computed densities *N* are quite similar with the two approaches; the corresponding controls *V* show similarities but also some differences, most pronounced near the surface during daytime. There are two sources of the observed discrepancy: The first concerns whether the mortality is multiplied on the value function *U* (left column) or on the constant fitness *F* (right column). The magnitude of this difference stems from the spatiotemporal fluctuations in the value function *U*, which can be seen in Fig. 1. The second possible explanation of the difference is numerical: Noting that the velocity fields *V* differ mostly between the two runs in regions with low concentration of the players, it can be argued that the objective function is quite insensitive to the velocity fields in such regions. The Newton-type algorithm, which has been utilized in the optimization, may have stopped before a true stationary point was identified.

**Fig. 3:**
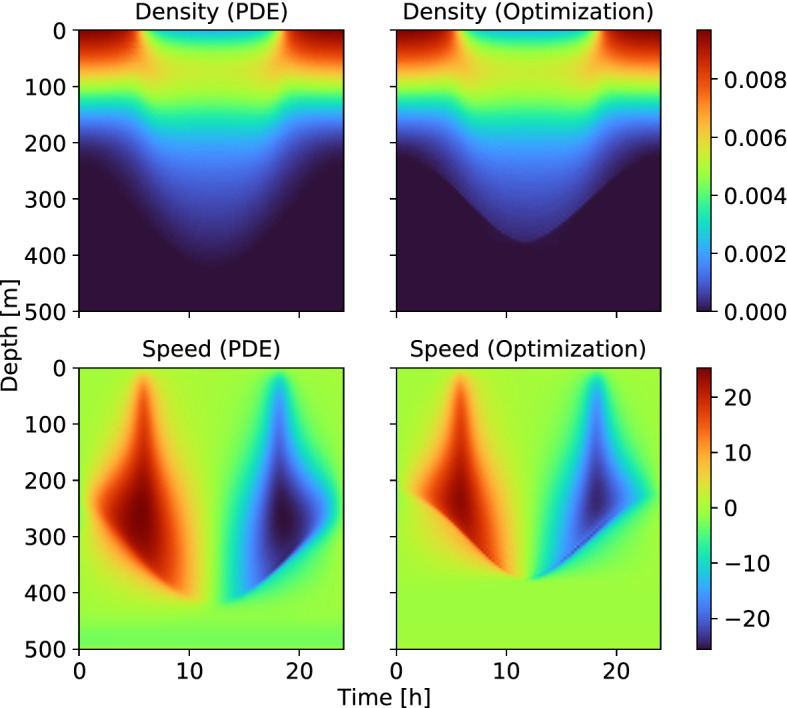
(Color figure online) Nash equilibrium for the second case with no low concentration regions in the optimal density. *Left column*: Mean field PDE system. *Right column*: PDE-constrained formulation

For the second test example, we select a parameter set without almost void regions near the surface in the optimal density *N*. We use the constructed example from “Appendix A” where the mortality scaling *m* have been reduced to make the region near the surface more attractive to the animals. The numerical solutions are shown in Fig. 3. Now, there are no regions near the surface with (almost) zero concentrations and no sharp transitions, and both methods provide almost identical solutions in this region. While the observed differences in the numerical solution can be explained as before—i.e. the quasi-static approximation and the convergence of PDE-constrained optimizer—the better agreement near the surface during daytime suggests that the optimization approach has bigger difficulties identifying optimal velocities in regions with very low abundances.

## Discussion

The starting point of this paper was our model proposed in Thygesen and Mazuryn (2022), and in particular, its limitation to the cases where there are no almost void regions—parts in a water column where the optimal density function has zero values. The issue comes from the fact that it is not clear how to properly define the vertical speed of a player in the nearly void regions without animals. We refer to set-ups with empty regions as the “deep water problem”.

In this paper, we have presented a novel model which addresses the issues with the zero concentration regions, covering deep water scenarios by including randomness to the model. Now, the players in the vertical game can adjust their vertical position by controlling their vertical speed with random disturbances in the state. The diffusivity ensures that there are, in fact, no strictly void regions. While the model in Thygesen and Mazuryn (2022) used the Euler–Lagrange approach, we have in here employed a Hamilton–Jacobi–Bellman formulation, in which it is well-defined what is the optimal vertical speed, even in regions where there are no animals—the optimal speed of a hypothetical agent which happens to find itself there even if it should not be there.

Diffusion terms in PDE models are common in ecology, see for example Okubo and Levin (2001), and randomness can correspond to errors in decision-making of the animals due to incomplete perception of the surrounding environment and emerge from search-type movements. This is reflected in the empirical fact that animals move unpredictably, but it can also result from structure of water flow, i.e. turbulence. In this latter case, though, one would expect correlation between neighbouring animals. It is important to emphasize importance of the assumption about independence of white noise processes for each player. Cases with correlated noise, as for the case with turbulence in the water column, lead to systems of stochastic partial differential equations which dramatically increase complexity of analytical and numerical tractability of the resulting models. This type of mean field differential games with common noise is not considered here and serves as a potential extension of the established results in this manuscript.

The model includes the density-dependent mortality term, which can be due to higher detection rate of animals in big groups. But this “group” interpretation is only one possible mechanism of the density dependence. As one alternative explanation of the mean field term is density-dependent foraging when a higher resource availability typically attracts higher concentration animals which results in a competition for the limited resource.

While deriving the final model, we take another approach than in our previous paper (Thygesen and Mazuryn 2022). Here, we employed the Feynman–Kac formula for establishing the equivalence between optimizing the fitness functional and the Hamilton–Jacobi–Bellman formalism. This allows us to include cases where the fitness fluctuates with time, so that the model framework can also be applied to situations where the environmental variations are not short compared to the life history of the animal. To our knowledge, this is the first explicit application of the Feynman–Kac formula in mathematical analysis within behavioural ecology, even if similar growth/mortality trade-offs are well-studied (Mangel and Clark 1986).

We disregard the source and mortality terms in the forward Kolmogorov equation due to the short time interval of interest, assuming that the players are long-lived. It is also a technical assumption which allows us to find a time-periodic solution to the Kolmogorov equation. As another potential direction of future research can be relaxing the periodicity assumption in the game and solving the mean field system with all the necessary terms in the Fokker–Plank equation.

We also establish the equivalence between finding a Nash equilibrium for the vertical differential game by solving the mean field system and the PDE-constrained optimization problem. These two approaches allow flexibility in the choice for a more suitable numerical framework for approximating a Nash equilibrium. We test these approaches on two examples and compare the final results. The two constructed examples provide consistent solutions in regions with nonzero concentration of the agents. Numerical issues arise in the optimization approach because the optimization problem is ill-conditioned, in the sense that the criterion is very insensitive to the strategy of animals in regions which are almost void. One way to address this inconsistency is to use heuristic procedures for estimation of a better guess or use more robust algorithms for numerical optimization. But one should keep in mind that the established equivalence is valid only under the quasi-static approximation, when fluctuations in the value function can be disregarded. Also the equivalence between the two formulations is valid for set-ups with a population of homogeneous players: the agents have symmetric functional and identical set of admissible strategies. This result does not directly translate to populations of heterogeneous agents where the symmetry assumption is relaxed.

To convert our mean field game to an equivalent PDE-constrained optimization problem, we must modify the density-dependent mortality from $\mu _1$ to $\mu _1/2$. In a mathematical study as the present, where specific parameter values are chosen for illustrative purposes, this makes little difference. Also, in empirical studies where density dependence is difficult to quantify, a factor 2 can easily be overlooked. The importance of this rescaling of density dependent mortality is mostly conceptual: It emphasizes that a mean field system, where agents pursue their own selfish interests, is inherently different from a system where agents pursue the common good, either through cooperation or through external regulation. To get the same solution, density dependence in the game must be stronger than in the system optimization. Stated differently, for a given density dependence, the game will predict stronger crowding than the system optimization. These observations are parallel to the similar discussion regarding the ideal free distribution (Fretwell and Lucas 1969).

As a potential direction for future work can be mentioned expansion of the modelling framework to several interacting populations to cover the multi-player set-up. In the current model, we only include implicit presence of the predators through the mortality term, but the model can be expanded to explicitly incorporate several vertical densities of a trophic network in the equations. Similar work with two populations has been done in Thygesen and Patterson (2019) but without taking into account the cost of motion. Another interesting direction to pursue might be, for example, if a population consist of almost identical animals, which however differ slightly in size, how do they partition the habitat among themselves?

One more possible direction for improvement is to omit the time-scale separation between the vertical game and population dynamics. This will lead to a mean field game formulation with population dynamics where the abundance of predators and preys are time varying quantities. Also, as mentioned earlier, for the sake of simplicity, our final model includes only vertical migration of the agents and disregards horizontal dynamics to avoid this level of generality and keep complexity to a reasonable level. It is also possible to include seasonal migration of animals to the model, when the agents can change their geographic location in the ocean.

To summarize, we have expanded the established PDE framework from Thygesen and Mazuryn (2022) to solve the deep water problem and cover all ranges of depth. A Feynman–Kac formalism has been utilized to derive the Hamilton–Jacobi–Bellman equation in the mean field game system. For the derived system of PDEs, we formulate the equivalent PDE-constrained problem under the quasi-static approximation and solve both formulations with the numerical scheme based on spectral elements method and validate the model results on several test examples.

## Figures and Tables

**Fig. 4:**
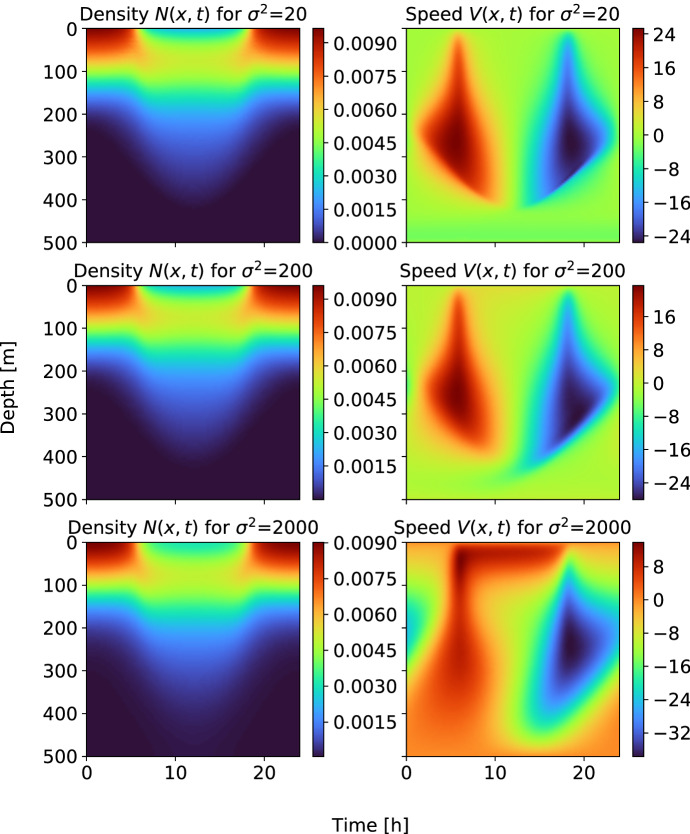
(Color figure online) Asymmetry manifestation in the vertical speed *V* for varying noise level $\sigma$

## Data Availability

Not applicable.

## Appendix A (A)symmetry of the Velocity Field

In this section, we elaborate further on the observed asymmetry in Fig. 1 in the optimal velocity field *V* with the symmetric optimal density *N* around the midday time. We discuss the observed feature of the velocity field in a more general set-up for an arbitrary fixed period $T > 0$.

To start with, we assume that the optimal distribution *N* is symmetric or an even function as a function of time:

A1 $$\begin{aligned} N(x, t) = N(x, T - t), \end{aligned}$$

for $$x \in [0, H]$$ and $$t \in [0, T]$$.

We aim to show that the symmetry of the density *N* in the sense (A1) does not imply that the vertical velocity field *V* is an odd function around on the interval [0, *T*]:

A2 $$\begin{aligned} V(x, t) = -V(x, T-t). \end{aligned}$$

To show that the equality (A2) does not hold, we differentiate the expression (A1) with respect to *t* and substitute the forward Kolmogorov equation (4) into both sides:

$$\begin{aligned}&\frac{\partial N}{\partial x}(x, t) = \frac{\partial N}{\partial x}(x, T-t) \\&- \frac{\partial (N V)}{\partial x} (x, t) + \frac{\sigma ^2}{2} \frac{\partial ^2 N}{\partial x^2}(x, t) = \frac{\partial (N V)}{\partial x} (x, T-t) - \frac{\sigma ^2}{2} \frac{\partial ^2 N}{\partial x^2}(x, T-t) \end{aligned}$$

Integrating both sides with respect to *x* from 0 to *x* and taking into account the zero-flux boundary condition (5) with the periodicity condition (A1), we arrive to:

A3 $$\begin{aligned} N(x, t) (V(x, t) + V(x, T-t)) - \sigma ^2 \frac{\partial N}{\partial x}(x, t) = 0 \end{aligned}$$

If (A2) holds, we reach the conclusion that $\partial N/\partial x = 0$. Thus, the velocity can only be symmetric if the optimal density *N* is constant in space, which would imply that the velocity profile *V* is zero everywhere. This argument relies on the assumption that $\sigma > 0$, and that we do in fact get symmetry in the absence of diffusion.

For the sake of completeness, to study numerically the behaviour of the vertical speed field, we take the parameters from Table 1 and vary the noise level $\sigma$. We also decrease the mortality scaling parameter *m* by 2 to avoid the low-concentration regions near the surface of the water column to eliminate high-speed values there and make the asymmetry more pronounced. Approximate solutions for the corresponding differential mean field game for various values of the diffusivity parameter are shown in Fig. 4.

It can be seen that for small values of $\sigma$, the speed field *V* approaches an odd function in the sense (A2) (right panel), while the qualitative structure of the surface of the optimal density *N* does not change across the parameter range, with only slight changes in the concentration of players (left panel). Slight differences in the optimal density for small values of $\sigma$ is due to the fact that the vertical migration is an advection-dominated process and contribution of the diffusion part is negligible. In contrast to small diffusivity values in the upper part of Fig. 4, in the lower plots with high noise level $\sigma$, we observe diffusion-dominated process with higher spread of the agents in the vertical game and more pronounced asymmetry in the optimal speed *V* as expected.

## Code Availability

The code used to perform the calculations and generate the figures will be placed in a publicly accessible GitHub repository after acceptance of the manuscript.

## References

[CR1] Aksnes DL, Utne ACW (1997). A revised model of visual range in fish. Sarsia.

[CR2] Alnaes MS, Wells GN (2015) The FEniCS Project Version 1.5. Arch Numer Software 3

[CR3] Bensoussan A, Huang T, Laurière M (2018) Mean field control and mean field game models with several populations. arXiv:1810.00783

[CR4] Bertucci C, Bertucci L, Lasry JM et al (2020) Mean field game approach to bitcoin mining. arXiv:2004.08167

[CR5] Brierley AS (2014). Diel vertical migration. Curr Biol.

[CR6] Carmona R (2016). Lectures on BSDEs, stochastic control, and stochastic differential games with financial applications.

[CR7] Carmona R (2020) Applications of mean field games in financial engineering and economic theory. arXiv:2012.05237

[CR8] Carmona R, Delarue F (2018). Probabilistic theory of mean field games with applications I: mean field FBSDEs, control, and games.

[CR9] Carmona R, Delarue F (2018). Probabilistic theory of mean field games with applications II: mean field games with common noise and master equations.

[CR10] Carmona R, Zhu X (2016). A probabilistic approach to mean field games with major and minor players. Ann Appl Probab.

[CR11] De Reyes JC (2015). Numerical PDE-constrained optimization.

[CR12] Fretwell SD, Lucas HL (1969). On territorial behavior and other factors influencing habitat distribution in birds. Acta Biotheor.

[CR13] Gabriel W, Thomas B (1988). Vertical migration of zooplankton as an evolutionarily stable strategy. Am Nat.

[CR14] Gomes DA, Nurbekyan L, Pimentel EA (2015). Economic models and mean-field games theory.

[CR15] Iwasa Y (1982). Vertical migration of zooplankton: a game between predator and prey. Am Nat.

[CR16] Kac M (1949). On distributions of certain wiener functionals. Trans Am Math Soc.

[CR17] Lasry JM, Lions PL (2007). Mean field games. Jpn J Math.

[CR18] Liberzon D (2011). Calculus of variations and optimal control theory.

[CR19] Mangel M, Clark CW (1986). Towards a unifield foraging theory. Ecology.

[CR20] Oksendal B (2013). Stochastic differential equations: an introduction with applications.

[CR21] Okubo A, Levin SA (2001). Diffusion and ecological problems: modern perspectives.

[CR22] Pinti J, Visser AW (2019). Predator–prey games in multiple habitats reveal mixed strategies in diel vertical migration. Am Nat.

[CR23] Rudin W (1976). Principles of mathematical analysis.

[CR24] Sainmont J, Thygesen UH, Visser AW (2013). Diel vertical migration arising in a habitat selection game. Theor Ecol.

[CR25] Thygesen UH (2022). Stochastic differential equations—an introduction for science and engineering.

[CR26] Thygesen UH, Mazuryn M (2022). Ideal free flows of optimal foragers: vertical migrations in the ocean. Theor Ecol.

[CR27] Thygesen UH, Patterson TA (2019). Oceanic diel vertical migrations arising from a predator–prey game. Theor Ecol.

